# Emerging applications of riboswitches – from antibacterial targets to molecular tools

**DOI:** 10.1007/s13353-016-0341-x

**Published:** 2016-03-28

**Authors:** Piotr Machtel, Kamilla Bąkowska-Żywicka, Marek Żywicki

**Affiliations:** 1 Department of RNA Biology, Institute of Bioorganic Chemistry, Polish Academy of Sciences, Z. Noskowskiego 12/14, 61-704 Poznań, Poland; 2 Department of Computational Biology, Institute of Molecular Biology and Biotechnology, Faculty of Biology, Adam Mickiewicz University in Poznań, Umultowska 89, 61-614 Poznań, Poland

**Keywords:** Aptamer, Aptazyme, Gene expression regulation, Biosensor, Riboselector, Riboswitch

## Abstract

The ability to precisely regulate gene expression is one of the most important features of the living cells as it enables the adaptation and survival in different environmental conditions. The majority of regulatory mechanisms involve protein action, however, multiple genes are controlled by nucleic acids. Among RNA-based regulators, the riboswitches present a large group of specific domains within messenger RNAs able to respond to small metabolites, tRNA, secondary messengers, ions, vitamins or amino acids. A simple, accurate, and efficient mechanism of action as well as easiness in handling and engineering make the riboswitches a potent practical tool in industry, medicine, pharmacy or environmental protection. Hereby, we summarize the current achievements and challenges in designing and practical employment of the riboswitch-based tools.

## Introduction

Regulation of gene expression includes a wide variety of mechanisms used by cells to specifically increase or decrease the expression of gene products (RNAs or proteins). Every step of gene expression can be modulated, from transcription initiation through the RNA processing and finally to the posttranslational protein modifications. Gene expression control at the level of nucleic acids is largely prevalent and well known in nature. Such mechanisms often include direct interactions between DNA or RNA and proteins (e.g., transcription factors, activators or repressors). Many regulatory proteins act simultaneously as molecular sensors, where formation of ligand-protein complex allows for maintaining a strict control over gene expression in response to various environmental stimuli.

Some domains of mRNA also possess the ability to sense small molecules and evoke regulatory effect. Such structures are called riboswitches and are found in all three domains of life. Riboswitches are regulatory, non-protein coding elements within mRNA, able to directly bind small metabolites, uncharged tRNA, secondary messengers, or ions. Bacterial riboswitches of 17 different classes are discovered in 36 human bacterial pathogens that can be targeted for addressing the ever-growing need for new antibiotics (Breaker 2012).

The size of riboswitches ranges from 34 nt for pre-queosine riboswitch to approximately 200 nt for lysine riboswitch. Riboswitches are especially common in Gram-positive bacteria, where they control the expression of at least 4 % of genes (Lünse et al. 2011). However, they are also found in eukaryotes (Cheah et al. 2007) and most likely in archaea (Weinberg et al. 2010).

Riboswitches are successfully competing with proteins for high sensitivity and accuracy in gene expression regulation. Therefore there is a reason to believe that the RNA World organisms could have used similar RNAs before the emergence of enzymes and genetic factors made of protein. The characteristics of some riboswitches strongly support this hypothesis. Furthermore, some riboswitch ligands are proposed to be molecular relics from an RNA World (Benner et al. 1989).

### Riboswitches structure

**Fig. 1:**
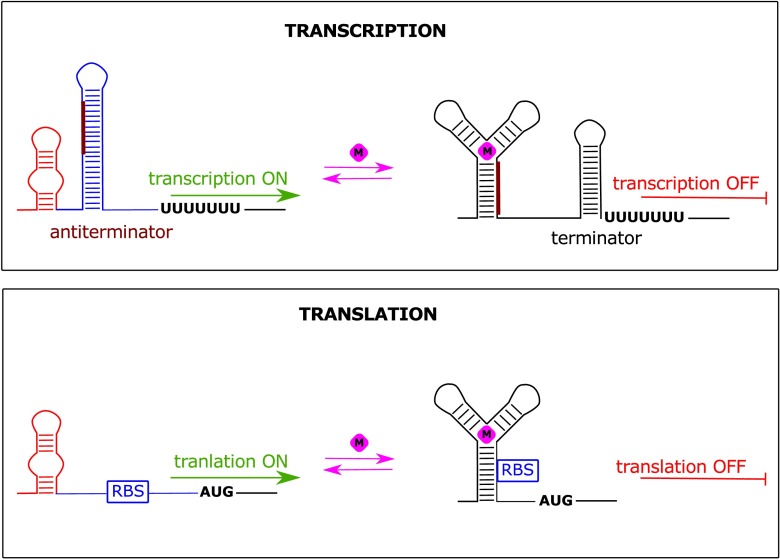
General mechanism of riboswitch action. The change in the structure of aptamer domain (red) triggered by metabolite binding (pink diamond) is transferred to expression platform (blue) causing switch in gene expression. The modulation of expression platform can cause the formation of transcription termination hairpin (terminator, upper panel) or differential accessibility of ribosome binding site (RBS, lower panel)

Structurally, riboswitches are composed of two regions: (i) evolutionary conserved aptamer domain, which is responsible for ligand sensing (presented in red in Fig. 1) and (ii) variable expression platform, controlling the gene expression (presented in blue in Fig. 1). Genes controlled by the concentration of a given metabolite are usually engaged in its biosynthesis, metabolism or transport.

A plethora of ligands binding to riboswitches, each recognized in accurate and specific manner requires a unique architecture perfectly matched to a given molecule. Additionally, different riboswitches belonging to one class may regulate the expression of more than one gene. For this reason, riboswitches reveal a huge diversity of structures. On the other hand, genes controlled by riboswitches are often involved in crucial metabolic pathways and cellular processes, thus the ligand-specific aptamer domains are usually strongly evolutionary conserved.

Based on structural features, riboswitches can be divided into two large groups: (i) pseudoknoted and (ii) junctional riboswitches (Serganov and Nudler 2013). In the case of pseudoknoted riboswitches, the RNA chain is predominantly folded into a single knot-like structure, composed of two stem-loops, where the part of one loop is engaged in base paring with the second loop. Such structures are found, among others, in pre-queosine, SAM-II and fluoride riboswitches (Gilbert et al. 2008; Klein et al. 2009; Li and Breaker 2012; Liberman et al. 2013). The ligand may interact with either the junctional region between helices or along the groove of the helix stabilized by pseudoknot.

The second group, the junctional riboswitches, is composed of a central loop playing a role of multihelical junction and several radial helices. The number of helices is variable and ranges from three for purine and thiamine pyrophosphate (TPP) riboswitches (Serganov et al. 2004, 2006) up to six for flavin mononucleotide (FNM) riboswitch (reviewed in Serganov and Patel 2009). The ligand binding site is usually located within the junction or regions adjacent to it, however, in some cases, the ligand may interact with distant regions of the riboswitch.

### Riboswitch-based regulatory mechanisms

Due to the architectural diversity, riboswitches can recognize a wide range of biologically important substances (for detailed review see: Peselis and Serganov 2014). The most abundant group comprises coenzymes and related molecules like: adenosylcobalamin (AdoCbl), thiamine pyrophosphate (TPP), flavin mononucleotide (FMN), S-adenosylmethionine (SAM), S-adenosylhomocysteine (SAH), tetrahydrofolate (THF), and molybdenum/tungsten cofactors (Moco/Tuco). The second largest group includes purines and their derivatives such as: guanine, pre-queuosine-1 (preQ1), deoxyguanosine (dG), cyclic-di-GMP (c-di-GMP), and cyclic-di-AMP (c-di-AMP). Furthermore, three riboswitches response to the presence of amino acids: lysine, glycine, and glutamine. One specific riboswitch binds phosphorylated sugar, glucosamine-6-phosphate (GlcN6P). The smallest ligands are metal ions: Mg^2+^, F^-^, and recently discovered Ni^2+^/Co^2+^ (Furukawa et al. 2015) and Mn^2+^ (Price et al. 2015). Apart from known and characterized riboswitches, there is also a group of so called “orphan” riboswitches-fully functional but without reported ligand (Meyer et al. 2011).

Riboswitches employ a wide repertoire of regulatory mechanisms. They may either silence or activate the gene expression, operating at the level of transcription, translation, mRNA degradation, splicing or RNA interference. The mechanism of riboswitch action is based on the existence of two mutually exclusive and thermodynamically stable conformations, separated by the energy barrier which inhibits the spontaneous switching (Fig. 1). In general, when the concentration of recognized molecule reaches the binding threshold value, the interaction with aptamer domain takes place. This promotes the switching to the alternative fold by stabilization of the intermediate and final states. The formation of riboswitch/ligand complex results in structural changes in the expression platform and consequently up- or down-regulation of gene expression.

The most frequent riboswitch-based regulation comprises transcription termination when in the presence of a ligand, the termination hairpin is formed. Therefore, in most cases, if the concentration of a ligand decreases in time, the anti-terminator structure is being formed within the expression platform by rearranging the conformation of one of the termination hairpin arms into the alternative structure (Fig. 1, upper panel). In such conformation, the mRNA expression is activated (Gilbert et al. 2006).

A portion of the riboswitches is involved in rho-dependent transcription termination. Rho factor is an essential transcription protein in prokaryotes with helicase activity. Rho binds synthesized RNA using its ATPase activity to provide the energy to translocate along the RNA until it reaches the RNA–DNA helical region, where it unwinds the hybrid duplex structure and prematurely terminates the transcription (Gilbert et al. 2006). Such mechanism is used by magnesium-sensing riboswitch in *mgtA* gene in *Salmonella enterica* or FMN riboswitch upstream *ribB* gene in *E. coli* (Hollands et al. 2012). In those cases, the presence of a respective ligand facilitates interactions with Rho helicases and contributes to premature transcription termination.

The mechanism of riboswitch-based translation control is primarily based on a similar system. However, the changes within the expression platform are related to the modulation of the ribosome binding site (RBS) accessibility instead of a termination stem formation (Fig. 1, lower panel). SAM-II riboswitch might serve as an example where the ribosome binding site is sequestered by the aptamer domain in the presence of SAM (Haller et al. 2011). In contrast, adenine riboswitch within the Add mRNA from *Vibrio vulnificus* is able to induce the protein synthesis upon the ligand binding by release of a Shine-Dalgarno sequence and a start codon (Reining et al. 2013).

One of the most interesting mechanisms for riboswitch-mediated gene control integrates ligand binding and ribozyme activities. A ribozyme is a ribonucleic acid enzyme that catalyzes a chemical reaction. Representatives of the glmS riboswitch class function as metabolite-responsive self-cleaving ribozymes, where GlcN6P serves as a cofactor for an autocatalytic cleavage of GlmS mRNA (Cochrane et al. 2007). After the cleavage, the mRNA is devoid of phosphate group at the 5’ end and therefore is susceptible to degradation by cellular RNase J, resulting in a reduction of GlmS mRNA level (Collins et al. 2007). An interesting example is the lysine riboswitch located upstream of *lysC* gene from *E. coli*, which presents a combined mechanism of gene expression regulation. The lysine binding causes the translation inhibition by modulation of the RBS accessibility. Simultaneously RNase E cleavage site becomes exposed, which promotes the degradation of a transcript (Caron et al. 2012).

Catalytic riboswitches can also be involved in regulation of RNA splicing. In bacteria, the known example is a c-di-GMP riboswitch in *Clostridium difficile*, which is located adjacent to the self-splicing intron in CD3246 mRNA, involved in bacteria virulence (Chen et al. 2011). In a presence of a c-di-GMP, a structural rearrangement promotes the choice of the splice site, which results in production of functional mRNA harboring the RBS. In contrast, when the concentration of c-di-GMP is low, the alternative 5’ splice site is selected, which subsequently leads to the exclusion of the RBS, thus preventing the expression of the protein.

Although the regulation of gene expression by riboswitches is generally a hallmark of prokaryotic organisms (especially Gram-positive bacteria), one example, namely the TPP riboswitch, was also found in eukaryotes. Usually, in the case of bacteria, TPP riboswitch operates at the transcriptional level (Bian et al. 2011). Eukaryotic TPP riboswitch controls the splicing and the stability of transcript instead. As a consequence, eukaryotic TTP riboswitches are located in introns or 3’UTRs of selected mRNAs (Cheah et al. 2007; Wachter et al. 2007). Introns may be excellent vehicles for riboswitch expression and regulatory function, and therefore may yield future riboswitch discoveries.

### Riboswitches as potential antimicrobial drug targets

**Fig. 2:**
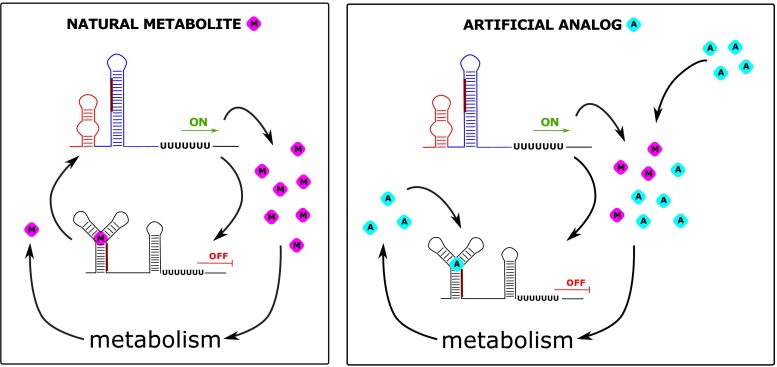
Mechanism of action of natural *versus* artificial antimicrobial compounds targeting riboswitches. Left panel: In most of the cases riboswitches are involved in the regulatory feedback loops by sensing the concentration of the metabolite (*pink* diamond) produced or imported into the cell by proteins under their control. When the concentration of the compound is dropping due to use in cell metabolism, the protein expression is activated (ON). After rise of the metabolite concentration, expression is again suppressed (OFF). Right panel: The riboswitches are targeted by artificial analogs of the original ligands (*turquoise* diamond). In order to be effective, the analog should not be used as a substrate in cell metabolism, causing permanent stalling of the riboswitch in the bound state. In case of the perturbation of a typical feedback loop, such stalling will lead to inhibition of the metabolite synthesis or uptake leading to cell death

The ability of the riboswitches to precisely discriminate between different cognate molecules as well as their common existence in bacteria makes them a promising target for antibacterial drug therapy (Fig. 2). From a medical point of view this issue is crucial because of a continuously progressing problem of bacterial resistance against regular antibiotics. The potential application of riboswitches as novel antimicrobial drug targets has several important advantages over classic antibiotics. First, they demonstrate potentially lower toxicity due to the fact that riboswitches are not found in higher eukaryotes, including human. Additionally, a lot of them are controlled by small compounds, simple metabolites, easy to deliver into the system, but also to manufacture and modify. The possibility of developing the resistance by bacteria against the antibiotics targeted at riboswitches seems to be more restricted than in the case of commercial drugs. The presence of a single type of riboswitches in multiple bacterial genes makes a single mutation insufficient to neutralize antimicrobial effect.

To consider riboswitches in terms of a potential pharmaceutical therapy, first of all, the analogs of ligands have to be found. Next, the administration of such compounds should permanently induce riboswitches even in the absence of native ligands.

#### Purine riboswitches

A group of riboswitches potentially applicable in the medicine are guanine, adenine, 2’-deoksyguanosine, and prequeuosine (preQ_1_) riboswitches, collectively termed as purine riboswitches. The genes controlled by these riboswitches are usually engaged in transport and metabolism of purines and may induce or silence gene expression after activation (reviewed in Lünse et al. 2014). The aptamer domains of guanine and adenine riboswitches are very similar and the difference lies basically in one nucleotide, pyrimidine at the 74th position, able to form Watson-Crick interactions with the ligand (Gilbert et al. 2009). After determining the exact nucleotide, cytosine or uridine, the riboswitch recognizes guanine or adenine, respectively and C > U transversion alters specificity from G to A. The sequences flanking the 74th nucleotide do not play any important role in ligand recognition but are responsible for increasing specificity and affinity by stabilization of purine in proper position (Mandal and Breaker 2004).

Because purines are essential for bacterial survival, a lot of effort is made to discover suitable analogs. It has been proved that some of the rationally designed analogs can bind the riboswitch with comparable affinity as guanosine and some additionally decrease bacterial growth (Kim et al. 2009). One of such analogs, 6-N-hydroxylaminopurine, was observed to repress the reporter gene expression downstream of the guanine riboswitch, hence regulatory effect may be ascribed to riboswitch activity.

Another potential riboswitch-targeting compound is 2,5,6-triamino-pyrimidin-4-one (PC1) (Mulhbacher et al. 2010). This compound was tested for its antibacterial properties on 15 strains of Gram-positive bacteria. In the case of nine strains, an inhibition of growth was observed. In this group of bacteria, guanine riboswitch controls *guaA* gene encoding GMP syntetase, whereas the resistant strains employ a different mechanism of *guaA* regulation. Among PC1-responsive bacteria there are found, *inter alia*, dangerous for human *Staphylococcus aureus* and *S. eidermidis*. PC1 was also tested on a murine model of mastitis caused by *S. aureus* infection. The results showed four order of magnitude decreases in the number of viable bacterial cells after PC1 administration. Moreover, it was observed that the presence of a reducing agent like DTT improved the potency of the therapy, achieving antibacterial activity comparable to those of some antibiotics. The drug was also examined on dairy cows (Ster et al. 2013). However, despite the significant reduction of bacteria in milk, only 15 % of subjects completely recovered, which can indicate the future need of the optimization of the therapy.

#### Lysine riboswitch

The lysine riboswitch is usually situated upstream of *lysC* and *lysP* genes. The first gene encodes for aspartokinase II responsible for phosphorylation of aspartate in the initial step of lysine, methionine, and threonine biosynthesis pathways. The second gene encodes the lysine permease. The lysine riboswitch is one of the largest (~200 nt in length) and most complex (five stem-loops) riboswitches. The lysine is tightly enveloped in a binding pocket enabling the discrimination by the length of the amino acid as well as a terminal amine group of the side chain (Sudarsan et al. 2003).

As a result of the screening for lysine analogs, four initially promising antimicrobial compounds were selected: L-aminoethylcysteine (AEC) (Ataide et al. 2007), L-3-[(2-Aminoethyl)-sulfonyl]-alanine, L-4-oxalysine, and Dl-trans-2,6-diamino-4-hexenoic acid (Blount et al. 2007). These compounds have similar affinity to the aptamer domain in comparison to lysine and they were shown to repress the gene expression by riboswitch activation. The last three analogs showed about fivefold decrease in bacteria concentration after 6 hours of incubation in comparison to untreated culture. Additionally they were able to completely inhibit bacterial growth for nearly 24 hours. However, some studies showed that AEC might be incorporated into proteins in mammalian and bacterial cells, which might lead to their toxicity (Di Girolamo et al. 1986, 1990). Furthermore, there are AEC-resistant bacteria strains with accumulated mutations in lysine riboswitch aptamer domain. Apart from the lysine riboswitch, AEC binds as a primary target to the lysil-tRNA-synthetase (LysRS) in bacteria (Ataide et al. 2007). It explains the observation of elevated level of free, cellular lysine in the case of down regulated *lysC* gene, since AEC competes with lysine for binding LysRS. The existence of additional target for lysine analog, independent of the site where the resistance can emerge, indicates future direction of more promising drugs, designed to effectively bind both lysine riboswitch as well as bacterial LysRS.

#### Cyclic-di-GMP riboswitch

Cyclic-di-GMP is a major prokaryote signaling secondary messenger. Considering eukaryotic organisms, its presence has been reported only in one species of amoebas (*Dictyostelium discoideum*, Chen and Schaap 2012), which makes c-di-GMP a perfect candidate for an antibiotic in the context of human safety. This compound controls a variety of different functions like: virulence, adhesion, cell aggregation, and biofilm formation. The most tempting for researchers is the possibility to control the bacterial virulence *via* modulation of c-di-GMP metabolism.

There are two classes of c-di-GMP riboswitches. Class I employs a classical mechanism of translation or transcription termination of downstream genes (Sudarsan et al. 2008). In contrast, class II c-di-GMP riboswitches functions as allosteric self-splicing ribozymes. In this case binding of the ligand promote the formation of the splicing product containing functional SD sequence in vicinity of the start codon, leading to activation of gene expression (Lee et al. 2010). The level of ligand discrimination by class II riboswitches is lower in comparison to class I (Shanahan et al. 2011). For both classes, the ribose with 3’-endo conformation is important as it provides high affinity ligand binding.

An example of effectively binding analog of c-di-GMP is endo-S-c-di-GMP (Zhou et al. 2012). This analog shows stable cyclic conformation which is crucial for the recognition by riboswitch. However, due to the fact that it is characterized by fourfold lower affinity compared to the natural ligand, further functional studies or optimization have to be performed.

Another promising group of compounds which might mimic cyclic forms of di-GMP are their linear forms (Furukawa et al. 2012). As recently demonstrated in β-galactosidase expression assay, some of them are characterized by high affinity binding and strong induction of the riboswitch. However, on closer inspection, some ligand-unresponsive mutants also showed similar expression profiles, which suggests that the therapeutic effect of linear di-GMP cannot be attributed to the riboswitch activity only.

#### glmS riboswitch

The *glmS* gene is present in many groups of Gram-positive bacteria since it encodes the protein converting fructose 6-phosphate phosphate and glutamine to glucosamine-6-phosphate (GlcN6P). The resulting aminosugar plays a crucial role in bacterial cell wall biosynthesis (GlcN6P is a precursor of peptydoglycan), being simultaneously indispensable for bacteria viability. 5’UTR of this gene operates as both ribozyme and riboswitch, catalyzing its own excision, leading to degradation of mRNA by RNase J1 (Collins et al. 2007). The *glmS* riboswitch is predicted to exist in at least 18 Gram-positive organisms, therefore it would be desirable to provide compounds that target this riboswitch and exhibit antimicrobial activity.

In order to define crucial structural features and functional groups necessary for the recognition by *glmS* riboswitch, the library of potentially active substances has been constructed. Upon library screening, it has been noticed that the amine group is important for enzymatic activity of the riboswitch and phosphate group is essential for high affinity ligand binding (Winkler et al. 2004; McCown et al. 2011). High level of molecule discrimination posed a challenge for researchers to identify the functional analog able to specifically block the riboswitch in its bound state.

The final breakthrough has been made in 2007 when carba-GlcN6P was proposed as a potential medicine for *Staphylococcus auresus* infections treatment and this compound is a subject of pending patent application (U.S. Patent No. 20140066409 A1, 2014). Finding a potent drug able to cure *S. aureus* infection is of special importance given the fact that this bacterium is an example of multiresistant strain. In the case of carba-GlcN6P, it has been noticed that both, the capability of the substrate cleavage as well as the reaction rate constant was performed with similar efficiency compared to the native ligand. Also bacterial growth was reduced by threefold in comparison to the control culture and a twofold decrease in *glmS* gene expression level was observed (Lünse et al. 2011).

#### TPP riboswitch

Thiamine pyrophosphate (TPP) is a derivative of vitamin B1, thiamine, an important cofactor of many enzymes involved in sugar and amino acid metabolism. In the cytoplasm, thiamine is metabolized to TPP by enzyme thiamine pyrophosphatase which makes this molecule biologically active (Ontiveros-Palacios et al. 2008). TPP riboswitches are located upstream of the genes engaged in transport and biosynthesis of TPP. Binding of the ligand causes the genes repression, mainly by transcription attenuation. The recognition of the ligand occurs within two helices: one interacts with pyrophosphate; the second recognizes pyrimidine of TPP (Thore et al. 2006).

The first described TPP analog demonstrating antibacterial properties, was pyrithiamine (PT) (Sudarsan et al. 2005). It was shown that both extrinsic and intrinsic PT inhibits bacterial growth and this toxic effect was fulfilled *via* down-regulation of genes under control of a TPP riboswitch. The same studies also revealed a mechanism of resistance acquisition during the evolution by accumulation of point mutation, usually A to G, in aptamer domain to disrupt PTPP-riboswitch binding. In the case of *B. subtilis*, such mutations occur in the riboswitch upstream of the *tenA* operon which functions as thiaminase. Interestingly, *tenA* by breaking PTPP into two molecules enhances the resistance effect.

Apart from the PT there is a growing number of a wide spectrum of TPP analogs with a sufficient binding, like: oxythiamine (Thore et al. 2008), amprolium, 4-methyl-5-hydroxyethylthiazole (Winkler et al. 2002a, 2002b) or benfothiamine (Edwards and Ferre-D’Amare 2006). However, these compounds did not seem to be as effective as PT in the activation of the TPP riboswitch.

#### FMN riboswitch

FMN riboswitch controls the genes engaged in transport and biosynthesis of vitamin B2—riboflavin. In the cell, riboflavin is converted to FMN by riboflavin kinase and then to flavin adenine dinucleotide (FAD) by FAD synthetase. Both substances constitute essential coenzymes in multiple oxidation and reduction reactions, and play a central role in energy metabolism (fats, sugars, carbohydrates, and proteins). The recognition of FMN occurs in a binding pocket composed of two peripheral stem-loops by interaction *via* a phosphate moiety, flavin ring, the ribityl with the aptamer domain in riboswitch (reviewed in Serganov and Patel 2009). The binding entails structural rearrangements leading to sequestration of SD sequence, and consequently, repression of a given gene (Ott et al. 2009).

The analog of riboflavin produced by *Streptomyces davawensis*—roseoflavin—is the only known example of naturally existing antibiotic targeting the riboswitch (Lee et al. 2009; Ott et al. 2009). Roseoflavin, after its biosynthesis, is excreted to the environment inducing antibacterial effect in bacteria species containing FMN riboswitch, which as a consequence helps to gain an advantage in struggle for survival. In the cells, roseoflavin is converted to roseoflavin-5-monophosphate (RoFMN) and interacts with the FMN riboswitch. This ligand is characterized by higher binding affinity in comparison to native FMN but the pattern of recognition remains similar. The research conducted on resistant and sensitive strains showed that single nucleotide at position 61 in the aptamer plays a crucial role in ligand discrimination (Pedrolli et al. 2012).

### Riboswitch-based gene expression control systems

The conditional control of gene expression has been the focus of attention of scientists for years. Although several systems of gene expression regulation exist, the riboswitch-based system is still able to contribute new quality to this field. It is characterized by several interesting features. First of all, riboswitches are often activated by small and simple compounds reducing costs of exploitation of such a system, even in industrial scale (for example IPTG is a much more expensive inducer of *lacZ* system). Furthermore, most of the ligands are small enough to penetrate a bacterial cell wall and cell membrane, which means that administration to the medium is sufficient to evoke regulatory effects. It appears to be an advantage in comparison, for instance, to antisense strategy where the antisense oligonucleotides have to be administered to the cells in suitable vectors like liposomes. Variety of different riboswitches makes them a flexible tool to control a broad repertoire of different genes. They utilize a diverse spectrum of regulatory mechanisms, from transcription termination to splicing control. Moreover, ligand binding may induce both down- or up-regulation, depending on the context in which aptamer domain is placed, increasing flexibility and potential range of application.

#### Riboswitch-based control of Mycobacteria gene expression

Inducible gene expression systems are widely exploited to analyze the gene essentiality (reviewed in Judson and Mekalanos 2000), function and potential drug targets (Miesel et al. 2003; Payne et al. 2007). There are several systems for gene expression control, however, most of them are limited to Gram-negative bacteria or related species. The example of such bacteria, important from the medical point of view are mycobacteria, including *Mycobacterium tuberculosis* (Mtb) and related species functioning as a model of tuberculosis, like fish pathogen *M. marinum*, non-pathogenic *M. smegmatis*, and bovine strain *M. bovis* used for BCG vaccines. All those species are important for health care and hard to handle for molecular genetics due to slow growth rate and risk of infection (reviewed in van Kessel et al. 2008). There are regulatory systems available for mycobacteria, namely acetamide-inducible *Msmeg* acetamidase promoter (Brown and Parish 2006) or tetracycline repressor-based system (Ehrt et al. 2005). However, both of them require suitable proteins to elicit regulatory effect which makes the whole system complex, difficult to calibrate and precisely control.

In 2012 the artificial theophylline-responding riboswitch was constructed to control gene expression in a wide spectrum of Gram-negative and Gram-positive bacteria, including *M. smegmatis*, a model for studies on tuberculosis (Seeliger et al. 2012). The theophylline riboswitch-based system consists of a synthetic aptamer domain sensing theophylline and a mycobacterial promoter of total length of about 300 nt. This system was used to induce and repress heterologous protein overexpression reversibly, to create a conditional gene knockdown, and to control gene expression in a macrophage infection model. In the absence of theophylline, the riboswitch adopts closed conformation making RBS and start codon inaccessible for translation machinery. Binding of theophylline induces structural alterations of the riboswitch and translation can occur. In *M. smegmatis*, the riboswitch construct demonstrated the activation ratio of ~89 for β-galactosidase and ~65 for GFP expression. The activation operated in a dose-dependent manner. Similar results were obtained for *M. tuberculosis* though this species is supposed to be even more sensitive to theophylline. The fact which seems to be of great importance is that unlike existing systems for controlling gene expression in Mtb, the riboswitch does not require the co-expression of any accessory proteins: all of the regulatory machinery is encoded by a short DNA segment directly upstream of the target gene. Additionally, the potential application of the theophylline-responsive riboswitch system to animal models of infection would be facilitated by the fact that theophylline is an FDA-approved drug and well tolerated in mice and guinea pigs.

#### Riboswitch-based control of virus gene expression and replication

The discovery that some naturally occurring viruses have an intrinsic preference to lyse cancer cells leads to the concept of providing “oncolytic viruses”, that are viruses that preferentially infect and kill cancer cells (Kelly and Russell 2007). The discovery initiated numerous attempts to artificially improve oncolytic properties by engineering known viruses (Wong et al. 2010). Hence the focus on riboswitch-based expression control systems capable of induction of viral genes in a reversible way.

Recently, two genetically modified viruses have been engineered: adenovirus (AdVs) and measles virus (ssRNA virus) with riboswitch-controlled gene expression (Ketzer et al. 2014). The basic idea of such a strategy was that the control of transgene expression by replication-deficient AdVs and replication-competent OAds were achieved by insertion of an aptazyme, a synthetic ligand-dependent self-cleaving ribozyme, into the 5′- and/or 3′-UTR of the transcription unit. Upon productive infection of a host cell with adenoviruses, the viral genome is transcribed in the host nucleus, mRNA is translated in the cytoplasm, and virions self-assemble in the nucleus. With engineered adenoviruses the viral genome replication occurs only for replication-competent OAds in tumor cells. Then, the transgenes are transcribed in the host nucleus. The applied OFF switch, given by an aptazyme, allows for transgene expression in the absence of ligand (gene expression ON). Upon addition of the ligand (theophylline), the aptazymes can fold into an active conformation and mRNA self-cleavage occurs (gene expression OFF). The studies showed the effective shutdown of *E14* gene expression accompanied with approximately 200-fold reduced replication rate, triggered by the addition of theophylline into the medium. This effect has been demonstrated in several cell lines.

Positive results were also achieved for measles virus (Ketzer et al. 2014). Here, the same aptazymes were incorporated in 5’ and 3’ UTRs of *F* gene encoding the F protein responsible for fusion of the host and viral cell membrane after attachment of the virus particle, controlling viral entry into the cell and spreading. The experiment proved that addition of theophylline reduced viral particles progeny up to 50-fold after 48 h.

In conclusion, because of their small size and RNA-intrinsic activity, the aptazymes could be considered as an alternative for inducible promoters in eukaryotic gene expression control.

#### Riboswitch-based gene expression in cell-like systems

The complexity of eukaryotic expression systems often makes certain phenomena hard to be fully understood. Therefore, an interesting alternative could be artificial cell-like systems that mimic key features of life with defined components but simplified and much easier to operate and regulate.

In 2011 Martini and Mansy presented an example of externally stimulated RNA-controlled function in two artificial cell-like systems: vesicle and water-in-oil emulsions (Martini and Mansy 2011). They have used previously selected theophylline riboswitch and constructed a working system with the use of yellow fluorescent protein DNA as a genome with T7 promoter and *E. coli* RBS. As a result, upon riboswitch activation by the ligand, the RBS was exposed for translation apparatus. The production of a reporter protein was active only in the presence of theophylline. Such a clear demonstration of controlled gene expression in water droplets and vesicles adds experimental verification to the ongoing origin of life research looking at compartmentalization of functional units.

#### Riboswitches as riboselectors

Riboswitches could be considered as sensor-actuator hybrids that can control gene expression in response to intracellular metabolite concentration. These characteristics substantiate the need for development of a riboswitch-based selection devices in order to expedite the evolution of metabolite-producing microorganisms. This device was named “riboselector” and its function was to specifically sense inconspicuous metabolites (Yang et al. 2013). A riboselector is comprised of two modules: a riboswitch and a selection module. Riboselectors can therefore efficiently optimize metabolic pathways by linking the intracellular concentration of a particular metabolite to survival of the cell under selection pressure. The lysine riboselector used by Yang et al. consisted of a lysine riboswitch from *lysC* of *E. coli* and the selection marker *tetA. lysC* gene encodes lysine synthase, which after lysine binding reduces downstream gene expression (Jang et al. 2015). A selective marker—*tetA*—encodes a tetracycline/H+ antiporter. Dual-selection modes of TetA enabled cells that accumulate lysine in the cytoplasm to survive in the presence of toxic metal salts, such as NiCl_2_. The application of positive selection pressure (addition of NiCl_2_) enriched the bacterial culture in higher lysine producer. Bacterial clones producing the largest amounts of lysine were able to repress *tetA* gene expression efficiently, thus could survive and replicate. In this system, a 75 % increase of a lysine production level was achieved after four cycles of the selection process. As a conclusion, riboselectors can give a growth advantage to metabolite-overproducing strains by modulating the expression of a selectable marker gene.

### Riboswitches as biosensors

A biosensor is an analytical device, used for the detection of an analyte that combines a biological component with a physicochemical detector. There is a wide variety of biological compounds used as recognition elements, including enzymes, antibodies, receptors, RNA, DNA, organelles, tissues, and finally whole cells (Han et al. 2010; Arora 2013). Recognition of a given molecule usually triggers a release of electrochemical or optical signal collected and quantified by the detector (Arora 2013). The most commonly used type of biosensor is an enzymatic one; nevertheless, the production of a high amount of enzymes is expensive and requires meticulous purification (Su et al. 2011). Hence, the scientific efforts were focused on immobilized whole-cell biosensors as they are characterized by low cost and improved stability in comparison to enzyme-based biosensors (Park et al. 2013).

**Fig. 3:**
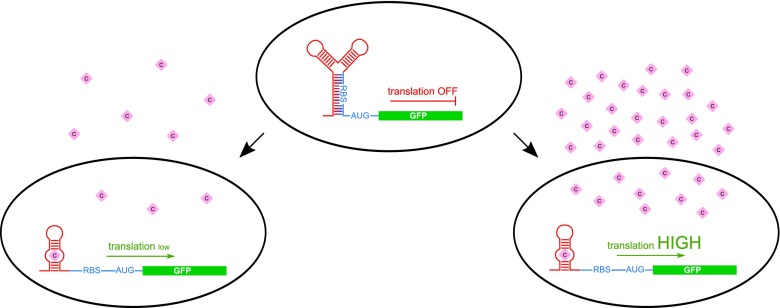
Employment of riboswitches as biosensors. The most common is the fusion of the specific artificial aptamer domain selected for a binding of a compound of interest (purple diamond) with the expression platform and a coding sequence of GFP protein. Such construct is introduced into bacterial cells. Due to the concentration-specific action of riboswitches, the transformed cells produce different amounts of GFP depending on the compound concentration within the cell (left *vs* right panel). Thus, the concentration of a compound could be estimated *via* GFP fluorescence measurement of the bacterial culture

Riboswitches offer a new promising approach to the biosensor field. The sensing ability of riboswitches (that is the sequence of the aptamer domain) might be generated *de novo* according to the needs by an *in vitro* selection process known as Systematic Evolution of Ligands by EXponential Enrichment (SELEX). This experimental procedure allows us to obtain the aptamer domain of a riboswitch responding to almost any small compound. Such artificial riboswitches, similarly to natural ones, bind ligands even in very low concentrations in a dose-dependent manner (Fig. 3).

In 2009, an anti-asthmatic drug theophylline biosensor was developed (Jo and Shin 2009). Monitoring of theophylline concentration in the blood is of special importance since its overdose can be dangerous for health causing seizures and heart rhythm disturbances (Pernites et al. 2011). The created biosensor was composed of a theophylline binding aptamer domain (serving as a sensing element) linked to the 5’ end of GFP coding sequence. The interaction of theophylline with the riboswitch caused a dose-dependent induction of GFP expression detected and translated to quantitative digital data by the signal transducer.

### Riboswitch-based control of bacterial behavior

Achievements of modern bioengineering in the field of microbiology allow us to control not only bacterial gene expression but also cell behavior, especially in terms of cell motility. Bacteria are naturally adapted to sense and respond to chemical signals from the surrounding environment. The basis of the bacteria chemical responsiveness is chemotaxis, the ability to recede or follow a given molecule according to its gradient in the habitat. Usually, a given chemical compound is recognized and bound by the surface receptor which causes the activation of the cascade of cytosolic proteins able to control flagellar motor complex (reviewed in Baker et al. 2006). It allows bacteria to avoid harmful substances and occupy habitats rich in nutrients.

Generally, the possibility to regulate bacterial mobility would be beneficial in a variety of different fields, like environmental protection to enhance bioremediation, targeted therapy or in performing complex tasks which require cooperation of more than one bacteria strain. In 2007 Topp and Gullivan showed that the *E. coli* chemotaxis system could be reprogrammed by placing a key chemotaxis signaling protein cheZ under the control of a theophylline-sensitive riboswitch (Topp and Gallivan 2007). Reprogrammed cells migrated up gradients of this ligand and autonomously localized to regions of high theophylline concentration, which is a behavior that cannot be accomplished by the natural *E. coli* chemotaxis system.

## Conclusions

Discovery of the riboswitches more than ten years ago gave scientists completely new possibilities of gene expression control within the cells (Table 1). Just as natural riboswitches can regulate gene expression in response to small-molecule ligands during transcription or translation, synthetic riboswitches can be engineered to repress or activate gene expression in a ligand-dependent fashion. Several interesting ideas have appeared so far to explore naturally existing or artificial riboswitches. The development of different analogs of native ligands finds potentially broad application in the field of medicine. Substances targeting riboswitches which control crucial bacterial genes might therefore constitute a novel potential source of antimicrobial drugs. The examples discussed herein reveal that increasing attention has been drawn to riboswitches and a number of intriguing compounds are available to date. Some of them have proven to be active in *in vitro* bacterial growth assays, but only one, targeting the guanine riboswitch of *S. aureus*, has been taken into animal studies so far. Up to date practical use of such compounds is limited by two major factors: (i) it is hard to create a compound which would retain the binding affinity of the natural ligand and would not be incorporated into host metabolic pathways and (ii) in most of known cases, the antimicrobial effect of the riboswitch targeting is caused by down regulation of a single gene or operon by a single riboswitch, enabling a relatively fast acquisition of resistance by bacteria.

**Table 1:** Summary of potentially applicable riboswitches

Ligand	Gene	Potential application	Reference
Lysine	*lysC*	Antibacterial drug target	Blount et al. 2007
Lysine	*lysC*	Riboselector	Yang et al. 2013
Glucosamine-6-phosphate	*glmS*	Antibacterial drug target	Lünse et al. 2014
Purines	*guaA*	Antibacterial drug target	Mulhbacher et al. 2010
Flavin mononucleotide	*ribB*	Antibacterial drug target	Pedrolli et al. 2012
Thiamine pyrophosphate	*tenA*	Antibacterial drug target	Sudarsan et al. 2005
Theophylline	-	Gene expression control	Seeliger et al. 2012
Theophylline	*E1A*	Virus gene expression and replication control	Ketzer et al. 2014
Theophylline	-	Gene expression in cell-like system	Martini and Mansy 2011
Theophylline	*CheZ*	Control of bacterial cell behavior	Topp and Gallivan 2007

On the other hand, possible modifications of riboswitch sequences and fusion of different aptamers with genes of interests pave a completely new way for generation of inducible expression systems and biosensors. It seems that in this field the major limitations have been overcome. Currently, there are efficient SELEX-based protocols available for development of aptamers specific for a wide range of molecules. Therefore, synthetic riboswitches can be employed to regulate the expression of any gene in response to any non-toxic molecule that is capable of being bound by RNA. This feature should enable RNA switches to play an increasingly important role as chemical biologists seek to modulate many types of cellular behavior in response to a broad range of chemical signals. What is more, apatmers used as conditional gene expression regulators or biosensors seem to provide both efficiency as well as robustness.

The ideas of practical riboswitch application presented in this article certainly do not exhaust the whole spectrum of possibilities. A number of further applications await their discovery, for development and implementation into practice. Nevertheless, the future of riboswitches as a molecular tool or antibacterial drug target seems to be genuinely promising.
